# Accuracy of remote diagnoses using intraoral scans captured in approximate true color: a pilot and validation study in teledentistry

**DOI:** 10.1186/s12903-020-01255-8

**Published:** 2020-09-25

**Authors:** Sabrina Steinmeier, Daniel Wiedemeier, Christoph H. F. Hämmerle, Sven Mühlemann

**Affiliations:** 1 Clinic of Reconstructive Dentistry, Center of Dental Medicine, University of Zurich, Zurich, Switzerland; 2 Statistical Services, Center of Dental Medicine, University of Zurich, Zurich, Switzerland

**Keywords:** Teledentistry, Remote diagnosis, Intraoral scan

## Abstract

**Background:**

Intraoral scans (IOS) provide three-dimensional images with approximate true colors representing a possible tool in teledentistry for remote examination. The aim of the present cross-sectional validation study was, therefore, to evaluate the levels of agreement between remote diagnoses derived from IOS and diagnoses based on clinical examinations for assessing dental and periodontal conditions.

**Methods:**

The test sample comprised 10 patients representing different clinical conditions. Following the acquisition of IOS (Trios, 3Shape), a full-mouth dental and periodontal examination was done and periapical radiographs were taken. Ten dentists were asked to perform dental and periodontal scorings for each of the ten patients on a tablet computer presenting the IOS. Scores included diagnosis of gingivitis/periodontitis, and evaluated presence as well as amount of plaque and calculus, and presence of teeth exhibiting gingival recession, furcation involvement, erosion, tooth wear, stain, and non-carious cervical lesion, as well as presence of decayed, filled, and crowned teeth and implants. In a second round of assessments, the periapical radiographs were provided and the dentists were able to change the scores. The time for the remote assessment was recorded. The agreement between remote and clinical scorings (reference) was then analyzed descriptively.

**Results:**

The mean time for the tele assessment was 3.17 min and the additional consultation of the radiographs accounted for another 1.48 min. The sensitivity and specificity values were 0.61 and 0.39 for gingivitis and 0.67 and 0.33 for periodontitis, with no relevant changes when radiographs were provided for the diagnosis of periodontitis (0.72 and 0.28). The agreement for dichotomized dental and periodontal indices ranged between 78 and 95%. With the provision of radiographs, the remote examiners were able to detect existing filled teeth, crowned teeth, and implants, whereas the detection of decayed teeth (70%) was not improved.

**Conclusions:**

The remote examination using IOS was effective in detecting dental findings, whereas periodontal conditions could not be assessed with the same accuracy. Still, remote assessment of IOS would allow a time-efficient screening and triage of patients. Improvement of the image quality of IOS may further allow to increase the accuracy of remote assessments in dentistry.

According to the Swiss Regulation this investigation is not a clinical trial and therefore no registration in a WHO-registry is needed.

## Background

Telemedicine is the remote delivery of health care services using information and communication technologies. The broad goal of telemedicine is to improve health of individuals and their communities in underserved areas [1]. Teledentistry is a sub-group of telemedicine and is successfully used within the dental practice for teleconsultation, telediagnosis and delivery of oral care services [2]. When access-to-care is limited (e.g. Covid-19 pandemic), teledentistry may allow the continuation of dental health services [3]. A systematic review concluded that teledentistry was a valuable tool for oral screening similar to face-to-face consultations [4]. Several electronic devices were used to capture the intraoral soft and hard tissues such as smartphones, digital extraoral cameras, or intraoral cameras.

Smartphones equipped with imaging technology are readily accessible and very easy to use [5]. A recent study during Covid-19 dissemination showed that patients could be successfully monitored using photos and a messaging service while reducing human contact [6]. In dental traumatology a clinical study showed that the remote diagnosis of dental traumas based on mobile phone pictures was similar to the diagnoses conducted in person [7]. A cross-sectional study showed, that smartphones with photo messaging can serve as an effective tool for the remote screening of potentially malignant oral disorders [8]. Standardized mobile phone pictures were remotely assessed for dental caries without radiographs in children with mixed dentition [9]. Sensitivity and specificity among several dentists were above 80%. A greater reliability was found at primary teeth as compared to permanent teeth. Similarly, a study showed that occlusal caries can be detected with acceptable diagnostic accuracy based on photographs taken by a smartphone camera compared to face-to-face screenings [10]. In orthodontics, the monitoring of linear tooth movements by means of a smartphone software showed an accurate assessment of the real tooth movements [11].

The use of small hand-held intraoral cameras was suggested as a feasible and potentially cost-effective option to a visual oral examination for caries screening in children [12]. A clinical study with a total of 62 children reported that the agreement between digital and conventional clinical examinations was very good for various oral conditions [13]. Sensitivity and specificity were 98.1 and 66.7% for caries. Similar values were obtained for the detection of stains (99% sensitivity; 77.8% specificity), calculus (98%; 72.7%), and tooth wear (90.3%; 81%). Another study reported that sensitivity and specificity were 73 and 98% for the remote screening of dental caries in young adults by means of five standardized intraoral photographs [14].

The diagnostic reliability in teledentistry may be limited to the image quality [15], missing clinical data [7], and the two-dimensional representation by photographs [13]. Today, intraoral scanners (IOS) are mainly integrated in the fabrication workflow of chairside reconstructions [16]. These devices can capture the intraoral conditions three-dimensionally and are augmented with close to true colors [17]. Also, IOS resulted in higher patient comfort and less chairside time compared to conventional impression techniques [18].

To the best of our knowledge, no clinical study has evaluated the validity of using intraoral scans with approximate true colors for the screening for diseases affecting the periodontal tissues and dental hard tissues. Evidence that IOS are valid for the screening may change the organizational structure of dental practices leading to a higher adoption and a wider distribution of this technology. Therefore, the aim of this clinical validation study was to assess the agreement between remote screening based on intraoral approximate true color scans and traditional clinical examinations.

## Methods

The study protocol was approved by the Ethical Committee of the University of Zurich, Switzerland, and categorized as a study not being regulated by the law on human research in Switzerland (BASEC-Nr. Req-2019-01277, 20.12.2019, retrospectively registered). Signed informed consent was obtained from the patients included in this study.

Patients in need of a prosthetic rehabilitation were consecutively screened at the Clinic of Reconstructive Dentistry of the University of Zurich, Switzerland, and recruited for the present study. The patients were examined by experienced dentists, who took the intraoral scans and recorded the intraoral findings.

An intraoral scan (Trios, 3Shape, Copenhagen) with approximate true colors was obtained in all patients. The scan included a full-arch scan of both jaws including all teeth and a bite registration of both sides in occlusion. The computed scans were issued with a separate study number to pseudonymize the intraoral scans before being sent to the store-and-forward based server (portal.3shapecommunicate.com).

Detailed dental records were obtained including number and location of teeth, location of caries lesions, of non-carious cervical lesions (NCCL), and of erosive lesions. The location, size, and material of fillings and restorations were also recorded. In addition, tooth mobility [19] and tooth vitality by means of CO2 testing were assessed. A full-mouth periodontal examination (FMPE) was recorded including probing depth, attachment levels, plaque index [20], bleeding on probing [21], furcation involvement, and gingival recessions. Periapical radiographs were taken when clinically indicated.

The clinical data for each patient were captured in a standardized assessment form and served as control (Table 1). In brief, the amounts of plaque and calculus were rated using a scale with 5 scores, whereas dichotomized scores were applied for the parameters gingival recessions, furcation involvement, tooth erosion, tooth wear, stains and NCCL. The existence of decayed, filled, and crowned teeth as wells as the presence of implants was checked. In addition, the quality of the restorations was evaluated. Gingival and periodontal health were evaluated using a scale with 3 scores (healthy, localized, generalized).

**Table 1:** Remote scoring form including overview of patient-specific clinical conditions (*P* = patient; *n* = 10 patients)

	Score	Criteria										
Clinical indices			P1	P2	P3	P4	P5	P6	P7	P8	P9	P10
Plaque	0	No plaque										
	1	Little amounts of plaque (less than 20% of the tooth surfaces covered with plaque)	x		x	x				x		
	2	Moderate amounts of plaque (less than 50% of the tooth surfaces covered with plaque)						x				x
	3	High amounts of plaque (less than 80% of the tooth surfaces covered with plaque)					x		x		x	
	4	Generalized plaque		x								
Calculus	0	No calculus				x						x
	1	Little amounts of calculus (limited to the lingual surfaces of the lower front teeth)	x		x		x	x	x	x		
	2	Moderate amounts of calculus (limited to the lingual surfaces of the lower front teeth and the buccal surfaces of the maxillary molars)										
	3	High amounts of calculus (involvement of more tooth surfaces than the ones of the lower front teeth and the maxillary molars)		x							x	
	4	Generalized calculus										
Gingival recessions	0	No recessions				x	x	x				
	1	Presence of recessions	x	x	x				x	x	x	x
Furcation involvement	0	No teeth with furcation involvement			x	x	x	x				
	1	Presence of teeth with furcation involvement	x	x					x	x	x	x
Erosions	0	No erosions	x	x					x	x	x	x
	1	Presence of erosions			x	x	x	x				
Tooth wear	0	No signs of tooth wear										
	1	Presence of tooth wear	x	x	x	x	x	x	x	x	x	x
Stains	0	No stains					x					
	1	Presence of stains	x	x	x	x		x	x	x	x	x
Non-carious cervical lesions (NCCL)	0	No NCCL					x					x
	1	Presence of NCCL	x	x	x	x		x	x	x	x	
Dentition												
Decayed teeth	0	No decayed teeth	x		x	x	x				x	
	1	Presence of decayed teeth		x				x	x	x		x
Filled teeth	0	No filled teeth										
	1	Presence of filled teeth	x	x	x	x	x	x	x	x	x	x
	1a	Sufficient (no intervention needed)										
	1b	At least one filling insufficient (need for intervention)										
Crowned teeth	0	No crowned teeth		x	x	x	x	x				
	1	Presence of crowned teeth	x						x	x	x	x
	1 a	Sufficient (no intervention needed)										
	1 b	At least one crowned tooth insufficient (need for intervention)										
Implants	0	No implants		x	x	x	x	x	x	x	x	x
	1	Presence of implants	x									
Diagnosis												
Gingivitis	0	No gingivitis										
	1	Localized gingivitis (less than 30% of the teeth involved)	x		x	x	x	x			x	x
	2	Generalized gingivitis (more than 30% of teeth involved)		x					x	x		
Periodontitis	0	No periodontitis			x	x	x	x				
	1	Localized periodontitis (less than 30% of the teeth involved)	x						x		x	x
	2	Generalized periodontitis (more than 30% of teeth involved)		x						x		

**Fig. 1:**
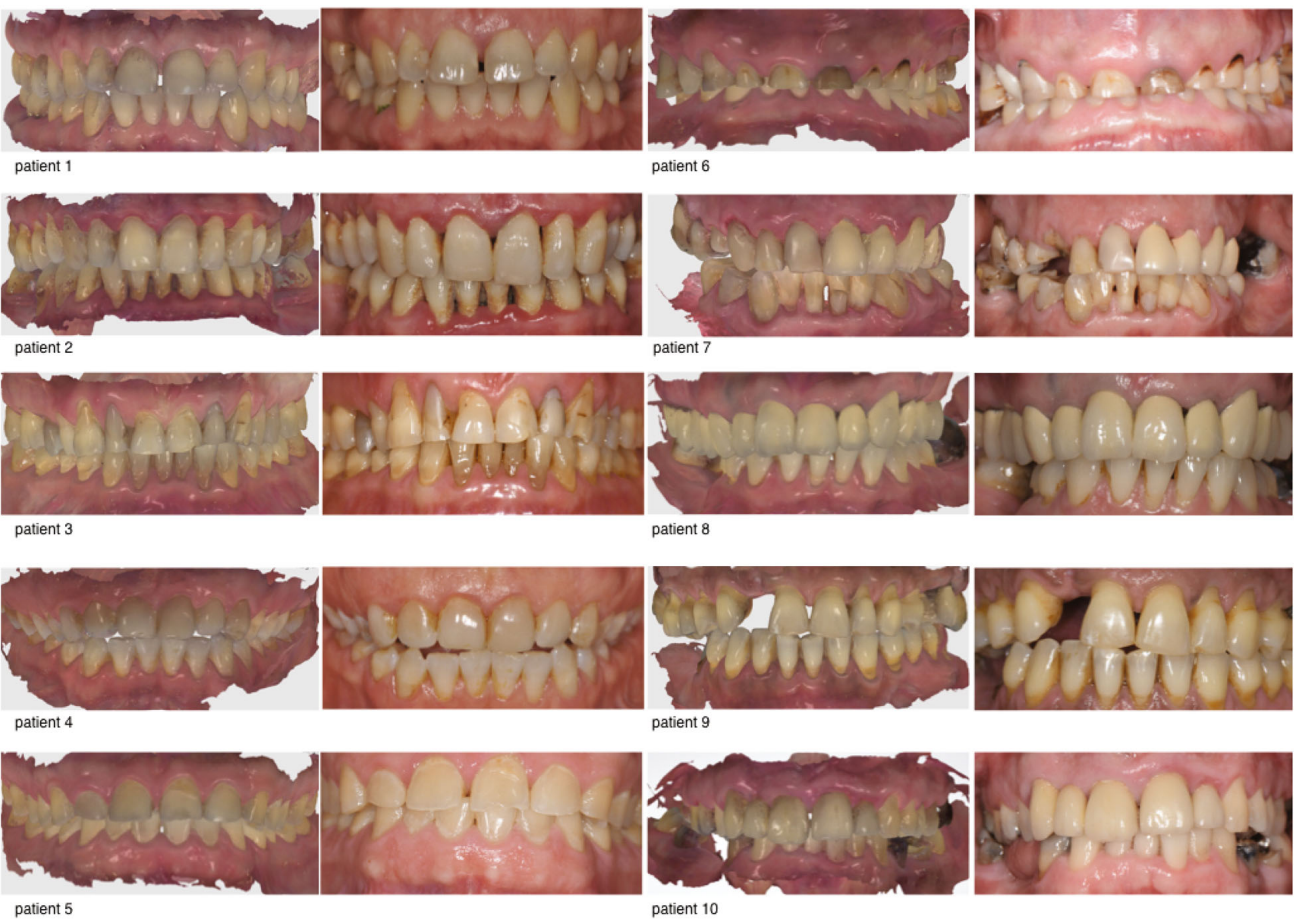
Patient overview (left IOS image, right intraoral photography)

For this validation study, ten patients were selected. The sample of patients was created to represent a wide variety of different clinical situations (Fig. 1). Edentulous patients and patients presenting with Kennedy class 1 were excluded. The overview of the clinical characteristics of the ten patients is presented in Table 1.

The remote examiners, who had a minimum of 3 years of experience as general practitioners, assessed the intraoral scans on a tablet computer (iPad Air, model A1474, Apple Corp. Cupertino, CA) using the software app provided by the manufacturer (3Shape Communicate, Version 4.0.1). The examiners used the standardized assessment form during the tele examination. Thereafter, they were provided with the periapical radiographs and asked to provide a second round of tele assessment. The time for the tele examination was recorded for assessment of the intraoral scans as well as for the additional assessment with the radiographs. The remote scoring (test) was compared to the scoring derived from the clinical data (control).

Each of the 10 patients was scored remotely by 10 examiners resulting in a total of 100 observations per parameter. Data was coded in Microsoft Excel. Analyses and illustrations were generated with the statistical software R [22], including the package ggplot2 [23]. For the parameters plaque and calculus dentists were grouped according to their years of clinical experience (less than 5 years of clinical experience versus more than 5 years of clinical experience).

## Results

The sample of examiners comprised a total of 10 clinicians (4 females, 6 males) with a mean age of 32.7 ± 3.5 years (range 28 to 41 years). None of the examiners has ever done a remote examination by means of approximate true color scans. The mean time (± standard deviation) for the tele screening was 3.17 (± 1.15) minutes and the additional consultation of the radiographs accounted for another 1.48 (± 0.59) minutes.

The agreement for the dichotomized clinical indices (gingival recessions, furcation involvement, tooth erosion, tooth wear, stains and NCCL) ranged between 78 and 95%. The presence or absence of decayed teeth was correctly assessed in 73% of the cases. This accuracy was higher for filled teeth (98%), crowned teeth (97%), and implants (96%). The provision of the radiographs allowed the remote examiners to correctly detect all filled teeth, crowned teeth, and implants, whereas the detection of decayed teeth (70%) was not improved. The false negative rate for the detection of decayed teeth was 21 and 23% with the provision of radiographs. The false positive rate increased from 3% without radiographs to 23% with radiographs.

**Fig. 2:**
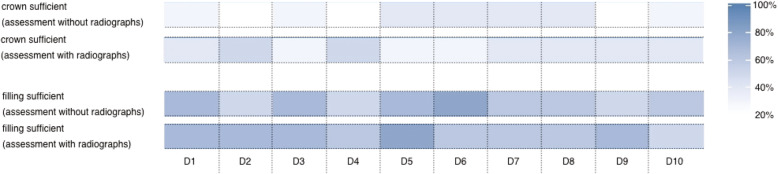
Heat map for the correct remote assessment of the sufficiency of fillings and crowns (D1 to 10; examining dentist, shades of blue represent the percentage of agreement)

The remote screening of the dentition for the presence or absence of insufficient fillings and crowns was correct in 63 and 65% of the cases and the provision of radiographs allowed to improve the accuracy for crowns (78%), but not for fillings (65%) (Fig. 2). The evaluation of the fillings resulted in 29% false negatives and in 7% false positives (31 and 4% with radiographs). The assessment of crown quality revealed 21% false negatives and 15% false positives with a decrease when radiographs were consulted (16% false negatives and 6% false positives).

**Fig. 3:**
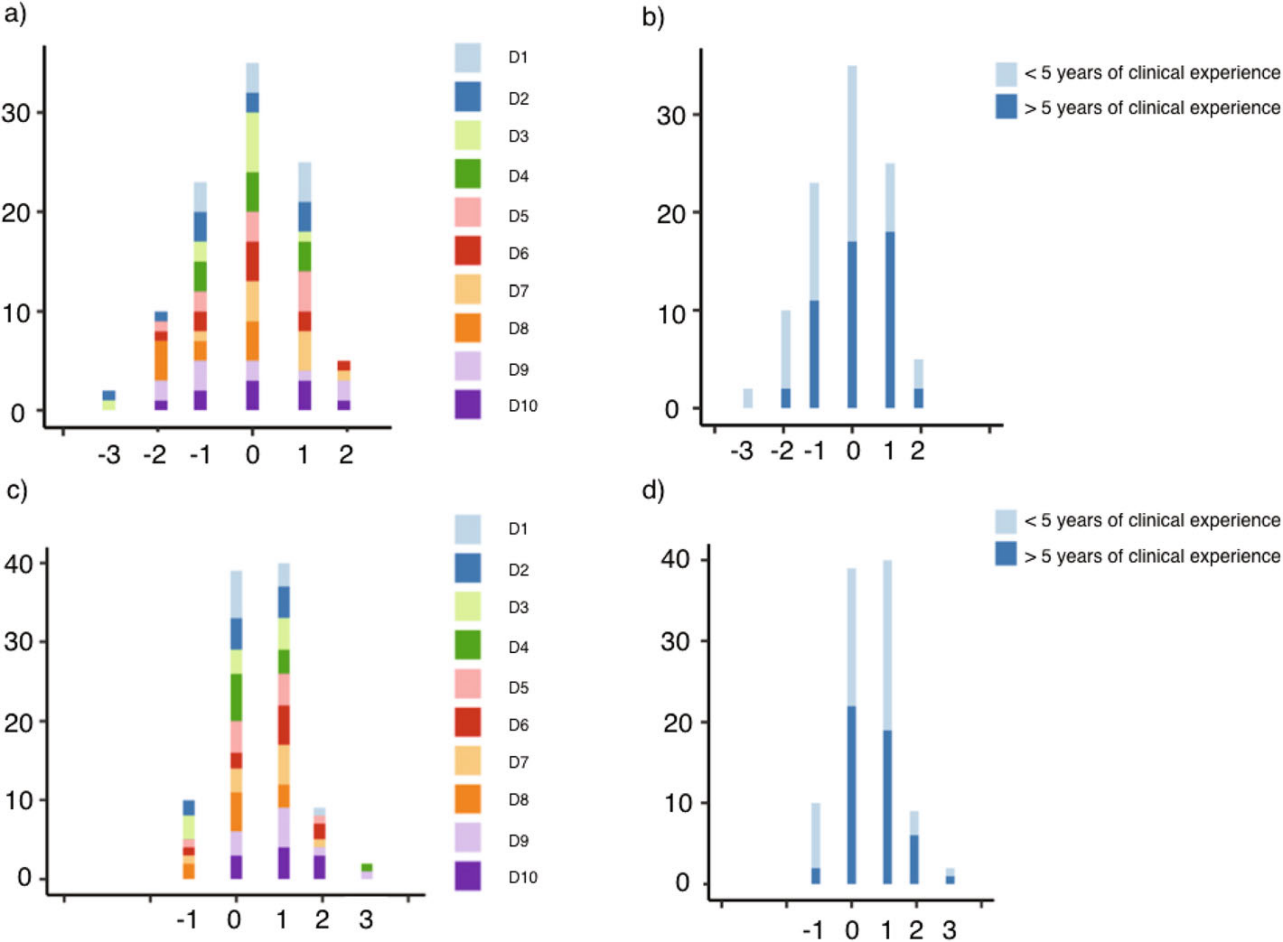
Difference in scores for the amount of plaque (**a**,**b**) and calculus (**c**,**d**) between remote and clinical assessment: 0 = perfect agreement, −/+ 1 moderate agreement, −/+ 2,3 poor agreement between remote and clinical score. (D, dentist)

The perfect agreement between the remote assessment and the clinical examination was 35% for plaque and 40% for calculus (Fig. 3). The tele consultation resulted in 35% higher plaque score values (overrating) and in 30% lower plaque score values (underrating) as compared to the clinical examination. The presence of calculus was overrated in 10% of the observations, whereas underrating encompassed 51% of the cases. A moderate agreement represented by a score deviation of ±1 unit was found for 48% of the plaque scores and 50% of the calculus scores, whereas a low agreement with score deviations of ≥2 units was limited to 17% (plaque score) and 11% (calculus score). The visual representation based on the clinical experience did not reveal a trend (Fig. 3).

The sensitivity and specificity values were 0.61 and 0.39 for gingivitis and 0.67 and 0.33 for periodontitis, with no relevant changes when radiographs were provided for the diagnosis of periodontitis (0.72 and 0.28) (Table 2). The false negative rate for gingivitis and periodontitis was 11 and 3%, respectively. The false positive rate was 28% for gingivitis and 30% for periodontitis.

**Table 2:** Frequency distribution of remote assessments for periodontal conditions (na, not applicable)

Diagnosis	Clinical reference	Remote assessments (*n* = 100)		Remote assessments with radiographs (*n* = 100)	
		correct	incorrect	correct	incorrect
no gingivitis	0	0	11	na	na
localized gingivitis	7	41	8	na	na
generalized gingivitis	3	20	20	na	na
no periodontitis	4	30	3	35	1
localized periodontitis	4	27	17	22	10
generalized periodontitis	2	10	13	15	17

## Discussion

The results of the present study showed that a dentist can make a time-efficient and valid tele screening of the oral soft and hard tissues based solely on approximate true color intraoral scans taken by means of IOS. The agreement between remote diagnosis and clinical diagnosis for dental and periodontal assessments, however, was moderate to low.

The continuous development of hard- and software has made it possible to electronically capture and transmit clinical pictures of patients. The accuracy of teledentistry, however, depends on the selected field of view. Two-dimensional intraoral images have their shortcomings, because they represent a limited two-dimensional view of three-dimensional soft and hard tissue structures. In a clinical study on the efficacy of remote screening for dental caries, up to 15.4% of all screened teeth could not be scored because of missing image data [24]). In the present study, all teeth were accessible for the remote screening because intraoral scans provide the full three-dimensional structure of teeth and gingiva. The fact that calculus scores were underrated, however, confirmed that only structures represented on the images can be remotely assessed.

In addition, the quality of the images may influence the outcome of tele evaluations. A clinical study providing smart phone pictures for the remote screening of potentially malignant disorders demonstrated that the false negative rate decreased as the camera resolution increased [15]. The results of the present study showed that the assessment of the remote score for the amount of plaque and calculus was limited. This may be related to the quality of the color images that the software uses for the colorimetric augmentation of the three-dimensional file. Anecdotal comments by the remote examiners suggested that the image quality on the tablet software may have been lower than in the IOS software emphasizing that hard- and software may influence the accuracy of remote screening.

The requirements of valid screening tools are time efficiency and accuracy. It was reported that the establishment of a FMPE took a mean of 29 min [25] and up to 40 min [26]. Therefore, tests and indexes were established to estimate the prevalence of periodontitis. A systematic review reported sensitivity values of partial-mouth examination protocols ranging from 57 to 96% with a higher sensitivity in protocols including the examination of all teeth [27]. The remote assessment of approximate true color scans including the gingival structures resulted in a sensitivity of 67% for the prevalence of periodontitis. More importantly, the false negative rate for periodontitis was very low (3%) demonstrating a low risk of missing out patients in need for a periodontal therapy.

A systematic review reported a range for sensitivity values by means of visual inspection for caries detection between 0.35 and 0.78 [28]. In the present study the remote inspection of intraoral scan data revealed a sensitivity of 0.88 for the detection of decayed teeth. The false negative rate for the detection of decayed teeth was high. Therefore, the present method cannot be recommended for caries screening. Recent IOS, however, are equipped with fluorescent or near-infrared light transillumination technology to detect caries lesions concomitant with the acquisition of the three-dimensional images. A clinical study showed the potential of this new technology [29]. Near-infrared light images allowed to detect proximal caries in posterior teeth similar to bitewing radiographs and were superior to the visual inspection alone.

The patient cohort of the present study was selected to represent a wide variety of different clinical situations. The low number of 10 patients, however, is a limitation of this pilot study. Also, comments by the remote examiners suggested that metal restorations were not properly represented on the approximate true color scans demonstrating a limitation of the IOS and its software used in the present study. The continuous development of the IOS will allow to overcome these limitations. Therefore, further clinical studies are needed to validate the diagnostic tools of current IOS.

The recording time as well as the investment costs for an IOS have to be considered. General dental practitioners showed concerns in relation to excessive time spent in capturing images and transmission of information [30]. The dental practices’ organizational structure, however, may be adapted by delegating image acquisition to dental auxiliary staff. Today, IOS are primarily used for the fabrication of reconstructions and dental appliances. The potential advantages associated with the use of IOS for remote screening of early diagnosis and the need for treatments may justify the high investment costs.

## Conclusions

The remote examination using IOS was effective in detecting dental findings, whereas periodontal conditions could not be assessed with the same accuracy. The use of a periodontal probe during clinical examination could not be compensated by IOS only. Still, remote assessment of IOS would allow a time-efficient screening and triage of patients.

## Data Availability

The datasets used and analysed during the current study are available from the corresponding author on reasonable request.

## Abbreviations

IOS: Intraoral scans
e.g.: For example
FMPE: Full-mouth periodontal examination
NCCL: Non-carious cervical lesions
